# Interventions to Improve Sexual Health in Women Living with and Surviving Cancer: Review and Recommendations

**DOI:** 10.3390/cancers13133153

**Published:** 2021-06-24

**Authors:** Jenna Sopfe, Jessica Pettigrew, Anosheh Afghahi, Leslie C. Appiah, Helen L. Coons

**Affiliations:** 1 Division of Hematology/Oncology/BMT, Department of Pediatrics, University of Colorado School of Medicine, Aurora, CO 80045, USA; 2 Center for Cancer and Blood Disorders, Children’s Hospital Colorado, Aurora, CO 80045, USA; 3 Department of Obstetrics and Gynecology, University of Colorado School of Medicine, Aurora, CO 80045, USA; jessica.pettigrew@cuanschutz.edu (J.P.); Leslie.appiah@cuanschutz.edu (L.C.A.); 4 Division of Medical Oncology, Department of Medicine, University of Colorado School of Medicine, Aurora, CO 80045, USA; Anosheh.afghahi@cuanschutz.edu; 5 Department of Psychiatry, University of Colorado School of Medicine, Aurora, CO 80045, USA; helen.coons@cuanschutz.edu

**Keywords:** female cancer, survivorship, sexual health, sexual function, body image, sexual desire, dyspareunia

## Abstract

**Simple Summary:**

Sexual health, both physical and psychological, is a common concern and unmet need among women with and surviving cancer. To guide clinical care and future research to improve sexual function and satisfaction in women with cancer, we performed a narrative review of interventions for sexual health concerns including sexual function, body image, genitourinary symptoms, and hot flashes. Relevant investigations conducted in the US and abroad published between 2005 and 2020 were reviewed (*n* = 91). Recommendations for future research in this area are also offered.

**Abstract:**

Sexual health concerns, both physical and psychological, are common and represent an unmet need among women with and surviving cancer. Sexual challenges and conditions negatively impact body image, satisfaction, relationships, well-being, and quality of life, yet are widely reported to be under-recognized and undertreated. To guide clinical care and future research on sexual function in women with cancer, we performed a scoping review of interventions for sexual health concerns, including sexual function, body image, genitourinary symptoms, and hot flashes. Relevant publications between 2005 and 2020 were identified by searching PubMed with a combination of medical subject headings and keywords. Articles were included if they focused on the aforementioned topics, were primary research publications, and included female cancer survivors. Studies focusing on women receiving hormone therapy for breast cancer were also included. A total of 91 investigations conducted in the US and abroad were reviewed. Most commonly, interventions included a component of psychoeducation, although pharmacologic, exercise, and other approaches have been evaluated. Many studies have focused on survivors of breast or gynecologic cancer, among other sampling and methodological limitations. These limitations underscore the need for more work on this vital survivorship issue. Recommendations for future research in this area are also offered.

## 1. Introduction

Sexual health, a key component of physical and emotional well-being and quality of life, is frequently negatively affected by cancer and its treatments. Unfortunately, sexual function concerns occur in 30–100% of cancer survivors, with one meta-analysis of female cancer survivors demonstrating a 60% prevalence rate [1,2,3,4]. Prevalence of sexual health challenges in female cancer survivors vary across cancer types and treatment modality, with gynecological cancer survivors experiencing the highest prevalence (78%), although prevalence is also quite significant among patients with breast and colorectal cancers (65% each) [4]. Beyond these populations, sexual issues span demographic characteristics including age and cancer type, with notably high prevalence also among childhood cancer survivors [2].

Consistent with a biopsychosocial model for sexual health, sexual challenges during and after cancer are present in varied ways depending on the impact of cancer, other physical conditions, and their treatment(s) and a patients’ psychosocial, mental health, trauma, and cultural histories [5]. Survivors experience a wide range of conditions, including arousal difficulties, vaginal dryness/atrophy, decrease in orgasm intensity or frequency, diminished sexual pleasure and desire, and dyspareunia, all of which can be complicated by and contribute to changes in body image and sexual-self-esteem [1,6]. Each of these aspects of sexual health may be affected by cancer and/or treatment, including systemic chemotherapy, targeted agents, immunotherapy, surgery, radiotherapy, and hormonal therapy. The pathophysiology varies, but includes anatomic, neurologic, and hormonal changes, such as a loss of sensation and premature menopause and psychological and social challenges related to cancer therapy and survivorship [1,2,7,8,9].

Unfortunately, despite the high prevalence and potential to affect the quality of life in cancer survivors, numerous studies demonstrate that such concerns are generally not addressed in women living with or surviving cancer [2,3,10,11,12]. Underdiagnosis and undertreatment may be attributed to communication barriers, a lack of time or prioritization, and a lack of provider knowledge/comfort with the topic, and, importantly, the challenge of treating such a complex physical and psychological issue [11,13]. It is critical to acknowledge that treatment for sexual conditions and issues in cancer survivors is unique to each patient, based on her specific concerns, cancer diagnosis, treatment(s), preferences, and relationship, sexual, mental health, and trauma history. Nonetheless, lessons for improving healthcare services for this important unmet need must be drawn from the scholarly literature. A scoping review is necessary for the following reasons: (1) A significant amount of research investigating new sexual health interventions has emerged in the past few years; (2) Several prior reviews focused on clinical recommendations and therefore had a different overall focus compared to this scoping review to define areas for future research [8,14,15,16]; (3) This review aims to inspire future research and therefore is not limited to randomized controlled trials [17], evaluating all recent interventional studies, inclusive of varied trial methods, sample size, or stage of research; (4) Since it may be possible to learn from intervention options across cancer types and sexual health conditions, and because addressing varied aspects of sexual health is critical in the biopsychosocial model, this review will not be limited by these factors, unlike some prior reviews [16,18,19]; lastly, future research should consider multimodal combinations of intervention types, this review is not limited by the type of intervention, as has been done in some prior publications [20,21]. As such, a scoping review was performed to summarize the current research evaluating interventions to improve sexual health (sexual function, body image, genitourinary and pelvic concerns, and vasomotor symptoms) in female cancer survivors and to highlight gaps in knowledge.

## 2. Materials and Methods

This scoping review summarizes published interventions to address concerns related to sexuality, including a range of sexual conditions, body image, sexual satisfaction, and menopausal symptoms in female cancer survivors.

**Figure 1:**
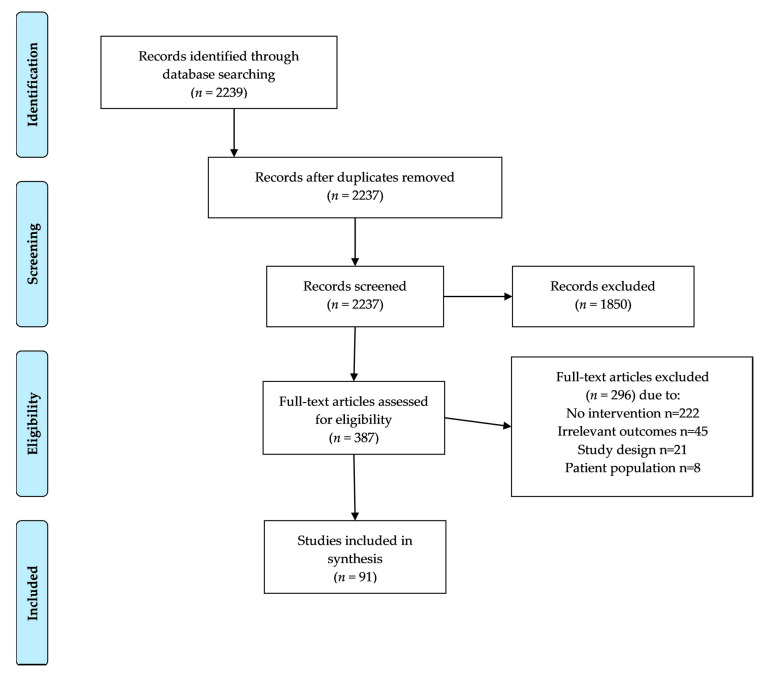
Flow diagram of identified and included publications.

A comprehensive literature search was performed in October 2020 with the following concepts: cancer, sexuality, sexual function, body image, and relationships and cancer survivors or survivorship (*n* = 2749). Relevant publications were identified by searching PubMed (via PubMed.gov accessed on 23 June 2021) with a combination of medical subject headings and keywords (Table S1). Additional articles known to authors were included (*n* = 3). Publications were limited to full articles, published in peer-reviewed journals from 2005 to 2020 and available in English (*n* = 2239) (Figure 1). As a scoping review, publications were included regardless of sample size.

Search results were compiled in EndNote, where duplicates were removed (*n* = 2). Covidence systematic review software (Veritas Health Innovation, Melbourne, Australia, available at www.covidence.org, accessed on 23 June 2021) was used for screening and full text review. The lead author screened titles and abstracts to address study criteria, excluding articles if they did not address the review question (e.g., focused on fertility or sexually transmitted infections), were not a primary publication, focused on on-therapy oncology patients (except for hormone therapy in breast cancer survivors), were limited to males, or were case reports, reviews, or a commentary. A data abstraction form was developed by the lead author, including the study method, intervention type, relevant outcomes (sexual function, body image, pelvic floor and vaginal health, and vasomotor symptoms) and findings, and notes. Publications were grouped by treatment indication and each author reviewed publications and completed the abstraction form relevant to her section.

## 3. Results

After limiting our review to articles published between 2005 and 2020, available in English, full text, and without duplicates, 2237 articles were screened. Of these, 1850 were excluded for lack of relevance based on the title and abstract, leaving 387 articles for deeper analysis. An additional 296 were excluded for various reasons (Figure 1), resulting in 91 articles included in this review.

### 3.1. General Sexual Concerns after Cancer

While sexual concerns after cancer are present in a variety of ways depending on the patient’s medical and social history, many studies have evaluated interventions to specifically improve sexual function and satisfaction, in general, in women or women and their partners. These are summarized in Table 1. The most common intervention approaches and those showing the most promise for a range of sexual health concerns are psychoeducational and psychotherapeutic programming. In this section, we explored these investigations by intervention type and cancer diagnosis.

#### 3.1.1. Psychoeducational and Psychotherapeutic Interventions

Most commonly, generalized interventions to improve sexual function and satisfaction have taken a psychoeducational, psychotherapeutic, or hybrid approach. Interventions most frequently combined psychoeducation about the impact of cancer and treatments on sexual health and symptoms, specific medical approaches and sexual health resources to treat sexual side effects, sex therapy techniques such as sensate focus to increase awareness of sexual feelings/sensations, cognitive behavioral therapy, hypnosis, mindfulness techniques to reduce negative thinking and distress, homework assignments, and follow-up telephone calls/booster sessions. In addition, some research focused on individuals while others targeted couples. The studies reviewed below to improve sexual health in women with cancer are organized in categories depending on how the intervention was primarily delivered (in-person or technology-based and in a group or individual setting), albeit almost all studies combined these approaches.

##### In-Person, Group Psychoeducational Interventions

Most of the in-person psychoeducational interventions have utilized group sessions, with promising results. For example, Rowland et al. conducted a randomized controlled trial (RCT) focusing on intimacy and partner communication in women with breast cancer [34]. The intervention included six two-hour psychoeducational groups aimed at improving sexual well-being in 83 predominately White women with moderate to severe concerns with body image, sexual function, or partner communication. Sessions focused on body image and sexual anatomy, sexual attitudes and behaviors, menopause, sexual functioning, and communication skills and homework. Four months after the intervention, relationship adjustment and communication and sexual satisfaction improved, especially among women who were least satisfied with their sexual relationship. Emotional functioning and sexual pain were not improved. De Almeida et al. found similar results in a smaller, but similar study, again in breast cancer survivors [38].

Ahmed et al. demonstrated that a similar intervention, specifically focused on younger women with breast cancer (45 years or younger), can be successfully delivered in a single day workshop [35]. While the study was small (*n* = 21) and lacked a control group, participants reported increased knowledge about sexuality and intimacy and especially greater knowledge of the techniques to increase sexual comfort after breast cancer.

One unique intervention targeted married Iranian breast cancer survivors (*n* = 50) and their husbands in a couples’ group intervention; both sexual function and sexual quality of life improved as a result of the intervention, compared to controls [36].

While the above studies are encouraging, the largest RCT (*n* = 194) had more modest results [37]. In this study, Esplen et al. tested an eight-week group program for breast cancer survivors (restoring body image after cancer (ReBIC)) combining guided-imagery to improve body image and self-perception in the future, group therapy support, education to improve body image, sexual functioning (e.g., vaginal dryness), and to address sociocultural factors impacting body shame based on a published self-image book [71]. At one-year follow-up, sexual function results were not statistically different between the intervention and control arm, although there was 25% improvement in sexual function scores in the intervention group but not the control arm. Reasons for differences between these results and other studies may include the intervention’s focus on body image (which did improve significantly), with sexual function as a secondary outcome, or the use of guided imagery, or the longer follow-up period compared to other studies. As with Esplen et al., another study using a psychoeducation model for Black or mixed Brazilian women (*n* = 23) with breast cancer yielded modest results, with sexual arousal but not sexual functioning improved with the intervention.

Psychoeducational and psychotherapeutic interventions addressing both biomedical and psychological aspects of sexual health may be particularly beneficial. Multiple studies have demonstrated that women with, or survivors of, ovarian and cervical cancer benefit from such combined biomedical/psychoeducational/psychotherapeutic interventions [49,56,57,58]. Several of these programs were delivered over a half-day with a single telephone follow-up. These programs included modules on education about treatment side effects, patient-provider communication, relaxation and body awareness, mindfulness-based CBT, individual problem-focused goals, and sexual symptom resources (e.g., moisturizers and dilators) [56,57,58]. Afiyanti, et al. delivered similar content to married Indonesian cervical cancer survivors (*n* = 53) over three sessions using a pre–post study design, with improvement in dyspareunia, satisfaction, lubrication, arousal, desire, and orgasm [49]. Importantly, a “sexual life reframing” group with psychological and biomedical components did not yield improvement in sexual health outcomes among married, Korean breast cancer survivors compared to controls (*n* = 45); this may be due to the sample size or, as authors noted, the use of sexual health questionnaires developed for a western population [33].

##### In-Person, Individually Delivered Psychoeducational Interventions

While most studies have involved group psychoeducation, a moderate number of individually delivered interventions have demonstrated improvement in sexual health outcomes. The largest of these was primarily education-focused and was conducted in 118 married Iranian women who underwent mastectomy for breast cancer [39]. This randomized intervention improved sexual function and quality of life, while satisfaction was not different between groups.

Other studies with similar interventions have demonstrated a more marked improvement in sexual and psychological functioning in predominantly White, menopausal women with rectal and anal cancers (*n* = 70) and Canadian endometrial and cervical cancer survivors (*n* = 31) [53,59].

Targeting a younger population, Canada et al. launched a pilot study to enhance the psychosexual development of adolescent girls (*n* = 12) and boys (*n* = 9) and young adults with, or surviving, cancer [28]. The intervention was similar to those above, although developmentally focused and offered in two sessions. Participants in the intervention group had more cancer-related sexual knowledge, fewer sexual concerns and less emotional distress at three months compared to the control group. Single participants were also less fearful to dating. These results adolescents and young adults with cancer are encouraging, but the findings were not reported by gender due to the small sample size.

Cieslak et al. studied the impact of a short trial of hypnosis on ten White, married women with breast cancer and one with breast and gynecologic cancer [40]. The uncontrolled, unblinded intervention included four in-person sessions, guided home practice, one follow-up telephone call at week five. Although limited by its small sample size, women subjectively reported positive changes in sexual satisfaction from week 1 to week 5.

While most in-person, individually delivered psychoeducation programs have been promising, the largest RCT, conducted by Chow et al., did not result in improved sexual health outcomes in 202 Chinese women with gynecologic cancers in Hong Kong, when compared to nurse-delivered general education [54]. In addition to typical psychoeducation, the use of herbs to promote recovery and gender roles in the Confucian beliefs was discussed. Results demonstrated improved outcomes in both study arms, with no difference between groups for sexual functioning or anxiety. The intervention group, however, which reported greater reductions in uncertainty with their illness, were more likely to be sexually active and reported that their partners had more sexual interest. The difference between this study’s more modest results and more promising outcomes in other investigations may suggest that this model is not superior to nurse-delivered education, or may be due to this interventions’ inclusion of women earlier in their treatment course (before treatment and up to 12 weeks after surgery), when there may be less capacity for improvement compared to later in, or following, acute treatments.

##### Technology-Based Psychoeducational Interventions

To improve the feasibility of and access to sexual health interventions, numerous studies have utilized telephone or online approaches, with or without in-person counseling. Five studies used telephone-based counseling for breast cancer survivors, with all but one demonstrating improvement with the intervention [41,42,43,44,45]. Importantly, two of these studies were large RCTs with sample sizes over 300 [42,44]. These two investigations and a smaller study by Reese et al. included a long (12 months to 5 years) follow up [45]. There are several unique differences between the five studies mentioned. While most interventions have been delivered by health care professionals, Schover et al. demonstrated in pilot and RCT trials that peer-delivered counseling results in improved sexual health knowledge and sexual functioning, and that there was no difference in benefits between in-person or telephone counseling [41,42]. Additionally, these two studies focused on African American women, unlike many other studies that have a predominantly White sample. It is important to note that the large RCT by Schover et al. did find that participants of both in-person and telephone counseling had improved sexual functioning at the six-month follow-up, although changes did not persist at 12 months [42]. Reese et al. used a unique approach of couples-based education and counseling (4 weekly sessions focusing on stress management, fatigue and sleep, diet and nutrition, and intimacy) for 29 breast cancer survivors and their partners, with positive changes in sexual and psychosocial (e.g., depression and anxiety) outcomes and sexual communication but not emotional intimacy [45]. In contrast, one single telephone-based couples intervention did not demonstrate a benefit, which the authors’ attributed to a lack of power (*n* = 26 dyads) [43]. Notably, though, couples did report satisfaction with the intervention. In addition, participants were given the option of telephone or in-person counseling, with no difference between these groups.

Several internet-based intervention studies were assessed. The largest of these tested a six-month program of weekly only CBT in an RCT of 169 breast cancer survivors in the Netherlands, with a three- and nine-month follow-up in 84 participants, compared to an information booklet alone [46,47]. The intervention group experienced improved sexual functioning, increased desire, arousal, vaginal lubrication, and pleasure and less pain during sex, less sexual distress, improved body image, and fewer menopausal symptoms post-treatment, which were mostly sustained at a three- and nine-month follow-up. The odds of improvement in sexual health following the intervention were 3.7 times more likely among the women with breast cancer who received the CBT intervention compared to the educational booklet only. However, changes in sexual satisfaction, psychological distress, relationship satisfaction, or health related quality of life did not differ across the two intervention groups. The positive impact of the CBT intervention on sexual health outcomes were maintained at three and nine months after treatment with the exception of sexual pleasure, which did not return to baseline levels [47].

Several other online studies used a more independent approach, with access to 12-week-long online self-help programs. Schover et al. created a sex-specific online self-help website for adult cancer patients and survivors, which was largely education-focused but included a component of CBT [26]. Notably, there was a high attrition rate (*n* = 60 of the original 197); participants who completed the program had a significant improvement in sexual function and increased sexual activity and use of sexual aids. Interestingly, sexual function improvement was not correlated with the level of website usage. It is possible that participants who will benefit will do so early and not feel compelled to return to the website; participants who do not benefit may continue to return to the website in the hopes of a benefit, and it might serve well to be directed to an in-person option once identified. Two other slightly smaller studies evaluated 12-week online self-help programs for breast, gynecologic, or colorectal cancer survivors [48,50], one of which tested this program in an RCT compared to the same program, supplemented by three in-person counseling sessions [48]. This RCT of breast and gynecological cancer survivors demonstrated significant improvement in sexual function and satisfaction, and reduced emotional distress across both groups, with more sustained changes in the group who also received the counseling sessions [48]. Importantly, this study was limited by a 22% attrition rate (initial *n* = 58) across both arms with younger women more likely to drop out. Brotto et al.’s single arm study of an online 12-week psychoeducational intervention for colorectal cancer survivors (with topics including sexuality and quality of life, sexual beliefs, anatomy and physiology, body image, mindfulness, communication, use of sexual aids, and more) demonstrated less sexual distress and genital pain, increased desire, arousal, orgasmic function, sexual satisfaction, and improved overall sexual functioning and mood following the intervention up to six months after participation [50].

These technology-based or hybrid interventions for women and couples facing breast cancer varied greatly in sample size and the intensity and length of the programs tested, but seemed to be well received, and were associated with similar or improved sexual health outcomes compared to control groups. Telephone, text, and internet approaches to service delivery may be especially effective in reducing geographic barriers for women living in rural and frontier areas, and more convenient for individuals who face challenges coming to an office for appointments because of poor physical well-being and/or competing work and caregiving responsibilities. In addition, some women and couples may find it more comfortable to learn about sexual health issues and treatment options in the privacy of their own homes. Furthermore, access to effective technology-based interventions to improve sexual function and satisfaction are vital during public health treats such as the COVID-19 pandemic.

##### Evaluation of Literature for Psychoeducational and Psychotherapeutic Interventions for General Sexual Function Concerns

US and international investigations to improve sexual health in women with varied cancers typically showed encouraging results for a range of sexual symptoms and psychosocial and relational outcomes. Positive changes were frequently reported regardless of whether the intervention was offered to individuals, couples, or groups, and whether delivered in-person or through technology. Unfortunately, conclusions and generalizations from this body of research are significantly compromised by methodological and sampling limitations. For example, many studies were not randomized to directly compare the intervention to controls, and significant variations in outcomes measured, duration of follow-up, and other important factors limit cross-study comparison. Further, because very few compared intervention formats (including in-person vs. online, individual vs. group, single vs. multi-session, and solo vs. partnered interventions), it is not possible to determine if a particular approach is more impactful. However, because programs across these types demonstrated promise, feasibility considerations might take precedence.

The studies also varied greatly about participant experience with cancer treatment. Some investigations recruited women in active hormone treatment while others had participants five or more years out from their diagnosis or treatment. Furthermore, studies were often limited to a certain cancer type (most commonly, breast cancer), early-stage disease, and partnered, middle aged women. Additional studies evaluating other cancer types, later-stage disease, and impacts of treatment modalities, in partnered and unpartnered women across the life span are necessary.

#### 3.1.2. Multimodal Sexual Medicine Clinics to Improve Sexual Health in Women with Cancer

While psychoeducational and psychotherapeutic programs can be very effective, women with physiologic and anatomic concerns may benefit from an interprofessional clinical approach to address their sexual concerns. To increase feasibility, a staged program, first through patients’ existing oncology or bone marrow transplant physician may be considered. For example, a pilot cohort study in hematopoietic stem cell transplant (HSCT) survivors (female *n* = 24) found that a monthly clinic conducted by a trained transplant physician resulted in improved sexual function, quality of life, and mental health [55]. This program included assessment, education and therapeutic interventions (vaginal estrogen, dilators, etc.), and, rarely, referral to a formal sexual health clinic. A different within-clinic program for survivors of all cancer types, which was nurse-led and consisted of a single visit only, qualitatively found participant satisfaction with the program and improved sexual well-being; quantitative measures were not significant, however this feasibility study was not powered to detect this difference (female *n* = 10) [22].

More formal sexual medicine services, staffed by a physician or advanced practice provider trained in sexual medicine or gynecology, and a psychologist with sex therapy expertise, have also been evaluated. Carter et al. describes one such program, which assessed sexual outcomes in 175 female cancer survivors who attended the clinic a mean of 3.4 times [23]. This program found high adherence to recommendations with significant improvement in vaginal symptoms and exam findings, sexual function across multiple domains, and increased sexual activity and confidence. Seaborne et al. also conducted a retrospective survey-based evaluation of their multimodal clinic. Almost 90% of their 113 predominately post-menopausal (68%) women with breast (57%) and gynecological cancers (32%) indicated that the service was at least somewhat helpful and that their sexual symptoms improved [24]. Qualitative evaluation of a similar multimodal program in a pilot setting found that gynecologic cancer survivors were satisfied with this approach and widely reported it met their needs [52]. While results of these programs are encouraging, studies utilizing objective, validated measures of sexual health symptoms, more varied cancer diagnoses, and evaluation of which program components are most beneficial are needed.

#### 3.1.3. Exercise Programs to Improve Sexual Health in Women with Cancer

Several exercise programs have been evaluated for their effects on sexual function, particularly among breast cancer survivors, with mixed but generally disappointing results. The largest of these was an RCT evaluating the effects of a year-long training and at-home exercise program for breast cancer survivors, including those on cancer-directed hormone therapy (*n* = 444) [29]. The intervention did not result in statistically improved sexual function at the 5-year follow-up. The long follow-up may have accounted for these results. Earlier outcomes may have demonstrated a difference between groups. Of note, a second report of the same study evaluated sexual activity using a different measure (*n* = 182), and the exercise intervention did result in increased sexual activity scores at the 5-year follow-up [30]. It is unclear if this was a natural change over time, as comparison to the non-intervention control was not reported. Similarly, a combined 8-week exercise and group psychotherapy program was evaluated qualitatively in 14 breast cancer survivors, without notable improvement in feelings of sexuality [31].

Among breast cancer survivors with, or at risk for lymphedema (*n* = 234), a year-long weightlifting program resulted in significant improvement in sexuality and romantic relationships [32]. There was no difference by lymphedema status, suggesting that this exercise program may be beneficial for all breast cancer survivors. Notably, this study did have a significant loss-to-follow-up (20%), with women who were lost to follow up having poorer baseline body image, relationship, and social support scores. Thus, the intervention itself may be less acceptable for the most at-risk group, which highlights a need to consider alternative and more tailored programming.

In a different population, one cohort study found that a six month-long personalized exercise program in endometrial cancer survivors (*n* = 63) was associated with a significant improvement in sexual function [51]. Improvement in sexual function was not correlated with amount of change in physical activity, although sexual interest was.

In summary, exercise programs have been assessed for their impact on sexual function, but studies have mostly been limited to breast cancer survivors and are also limited by study design, with only one RCT. Regardless, these programs largely did not have a significant impact on sexual function.

#### 3.1.4. Hormone Replacement in Women with Cancer with Low Sexual Desire

When treating postmenopausal women for decreased sexual desire, there is evidence for physiologic doses of transdermal testosterone in addition to systemic estrogen. However, there are no FDA-approved testosterone agents available for women. Barton et al. examined the use of physiologic doses of transdermal testosterone (10 mg daily) in post-menopausal women with a history of cancer who were not currently on cancer-directed hormone therapy [27]. In this randomized double-blind, placebo-controlled trial (*n* = 150), it was observed that while serum testosterone levels increased in the intervention group, there was no difference in sexual desire between the two groups. The authors hypothesized that this may be attributed to the fact that no participants were taking systemic estrogen, whether these women would benefit is not known. However, the safety of testosterone needs to be further investigated in all women, particularly women with a history of hormone receptor positive cancer, as it is converted into estrogen.

#### 3.1.5. Complementary and Alternative Medicine Practices to Improve Sexual Health in Women with Cancer

A large (*n* = 186) RCT was conducted to evaluate ArginMax in female cancer survivors, an over-the-counter nutritional supplement with extracts of L-arginine, ginseng, ginkgo, and damiana and multivitamins and minerals [25]. While sexual function scores were not significantly different between the intervention and control groups, quality of life was improved in the intervention group.

#### 3.1.6. Summary: General Sexual Function Interventions

In summary, there is reasonable evidence to suggest that broad-ranging sexual function concerns may be successfully addressed through combined psychoeducation, psychotherapeutic and hybrid or multi-component approaches, and through multidisciplinary sexual medicine programs. Notably, there is a scarcity of literature aimed at partner dyads; because sexual health is a biopsychosocial phenomenon, further research exploring the utility of addressing cancer survivors and their partners is necessary. Beyond this, many of these studies were focused on women with breast and gynecologic cancer. Additional research for a wider range of populations is of utmost importance. Further, interprofessional sexual medicine programs for women (or adults) with cancer are highly specialized and are predominately offered at academic health centers with major cancer programs. Telemedicine and other online platforms must be considered to extend effective sexual health interventions to women in varied communities. Importantly, online programming, while earlier in exploration, appears to be an effective and feasible way to deliver such interventions, and reduce barriers to care. In the absence of specialized sexual medicine programs for cancer survivors, utilizing more readily available specialty care through gynecology, sex therapy, and cognitive behavioral therapy is suggested. Lastly, exercise interventions, hormone replacement, and nutritional supplements have been evaluated, mostly in breast cancer survivors; these studies were largely disappointing with minimal or no effect on sexual function found. However, a few exercise interventions did demonstrate some improvement in sexual function, therefore, further evaluation with additional RCTs and broader patient populations may be warranted.

### 3.2. Body Image Concerns

Many cancer survivors experience changes in, or threats to, body image. Following invasive, intensive, or disfiguring treatments, diminished energy, changes in weight, hair loss, scarring, or anatomic changes, women may feel different about their body or sexuality [72,73,74] and attractiveness. Several intervention studies have aimed to address body image primarily or as a secondary outcome in relation with sexual function, these are detailed below and summarized in Table 1.

#### 3.2.1. Psychoeducational and Psychotherapeutic Programs

As in addressing general sexual function concerns, a variety of studies have aimed to improve the body image of patients after a cancer diagnosis through a combination of psychotherapy, mindfulness, and education. Most psychoeducation programs for breast cancer survivors, with one exception [33], showed benefit to body image at some time interval, from 3 to 12 months after completion of the programs [37,47,66,67,68]. Importantly, several of these programs successfully utilized group therapy [37,66,67,68], and several were successfully delivered online [47,68]. Notably, only two of these studies were moderate or large RCTs [37,67], so improvement in other studies could also represent natural improvement over time. While many of these studies were conducted in predominantly White, western populations, one of the studies with encouraging results was conducted in an Iranian population [67]. There was one psychoeducational intervention study that did not demonstrate a statistically significant improvement in body image; this study, which focused on Korean breast cancer survivors, may have its different results explained by its shorter follow-up, small study size, use of only a 3-item scale developed for a different cultural, and the intervention’s primary focus on sexuality [33].

Beyond breast cancer populations, one of the aforementioned group psychoeducation programs [37] (ReBIC, described in Section 3.1.1) was also pilot tested in gynecologic cancer survivors [68]. While this pilot study (*n* = 44) did not separate breast and gynecologic cancer survivors, body image improved across participants pre- to post-intervention, suggesting that such programming might be extrapolated to survivors of other cancer types. Graboyes et al. also reported that a pilot telemedicine-based cognitive-behavioral program for head and neck cancer survivors (*n* = 10) resulted in improvement in body image 1- and 3-months after the intervention [70]. Of note, this study was not able to separate female and male participants, but seven of ten participants were female. Lastly, a previously mentioned in-person psychoeducation study by Canada et al. (details found above), focusing on psychosexual development in adolescent and young adult (AYA) cancer survivors (ages 15–25) resulted in improvement in body image and overall decreased psychological distress (*n* = 21, females *n* = 12) [28].

In addition to traditional cognitive-behavioral therapy psychoeducation, mindfulness and relaxation techniques may help to address body image concerns in AYA cancer survivors (age 15–25; *n* = 21) [60]. Similarly, a 5-week hypnotic relaxation program demonstrated promise in a pilot study, which found qualitative improvement in body image among breast (*n* = 10) and gynecologic (*n* = 1) cancer survivors [40].

#### 3.2.2. Social and Expressive Programming to Improve Body Image

Social and expressive programming has attempted to target body image, self-esteem, and self-compassion among cancer survivors, particularly among the AYA population. Rosenberg et al. evaluated a week-long outdoor adventure program for young adult (age 15–39 years) cancer survivors [61]. Among patients who had identified cancer as adversely affecting their quality of life, there was significant improvement in body image after the program. However, study results also suggested that body image improvement may not endure over time, with participants who entered the program a second time having similar second pretest results to those who had not yet participated.

Both expressive writing and self-portraiture may result in improvement in self-esteem. Interestingly, a small (*n* = 10) pilot study of a two-session guided portrait experience in AYAs, self-esteem was improved, but body-esteem was not [63]; of note, because of the small sample size, data by sex was not reported. In contrast, body image, body appreciation, and self-compassion of breast cancer survivors improved significantly through a 30-min internet-based expressive writing program [62].

#### 3.2.3. Exercise Programs

As previously described, Penttinen et al. completed a large RCT of a year-long exercise intervention for women with breast cancer, with an evaluation five years after completion of the intervention [29]. Unlike sexual function, improvement in physical performance was correlated with significant improvement in body image and quality of life. Similarly, the previously reviewed program for breast cancer survivors with, or at risk for, lymphedema, found that a year-long weightlifting program resulted in significant improvement in body image, again, this study must be noted to have a 20% attrition rate [32]. A 12-week Pilates program piloted in 15 breast cancer survivors also demonstrated significant improvement in body image across most subscales and improvement in quality of life [65].

#### 3.2.4. Cosmetic Interventions to Improve Body Image Related to Skin Concerns

Women with breast cancer on antiendocrine therapy such as tamoxifen are at risk of skin changes that may affect body imaging. Dalenc et al. demonstrated success of a three-week skin hydrotherapy program for improving body image and quality of life in an RCT (*n* = 68) [64].

Among women with head and neck cancer, there may be significant cosmetic concerns related to surgical scars and radiation therapy. An in-depth skin camouflage program did not result in improved body image, but did notably reduce social anxiety in a small RCT (*n* = 66) [69].

#### 3.2.5. Summary: Interventions to Address Body Image Concerns

Similar to treating sexual function concerns, psychoeducational programming, whether group or individual and in-person or online, may be an ideal way to improve body image in cancer survivors of all ages. Again, while psychoeducational programming may address the social/relational aspects of sexual health, partner-focused interventions may also be beneficial; we did not identify any dyadic interventions aimed at improving body image. Exercise programs and cosmetic interventions may also be beneficial, but studies are limited. Again, most studies have focused on breast cancer survivors, necessitating future research to further establish utility of such programming for other populations of cancer survivors.

### 3.3. Genitourinary Syndrome Impacting Sexual Health in Women with and Surviving Cancer

Symptoms of vaginal pain and dryness, termed genitourinary syndrome of menopause (GSM), affect up to 70% of breast cancer survivors on ovarian suppression and survivors of other cancers who experience premature menopause as a result of surgery, chemotherapy, or radiation, and women who undergo pelvic radiation even without premature menopause [75]. With the cessation of ovarian function, the estrogen receptors in the urogenital tract are left unbound, which results in pallor, loss of elasticity, dyspareunia, and vaginal dryness. These changes may result in sexual pain and decreased pleasure, which may be accompanied by significant distress. Many women are not aware of how their cancer treatments will affect their genital tract and the sudden changes come as a distressing surprise. Treatment of these symptoms can be broadly categorized as physical therapy, nonhormonal topical agents (e.g., vaginal moisturizers and lubricants), hormonal topical agents, oral selective estrogen receptor modulators (SERMs), and procedures such as CO_2_ laser therapy (Table 2).

#### 3.3.1. Non-Hormonal Topical Agents for Genitourinary Syndrome

For women who have medical contraindications or prefer nonhormonal treatment, there are several options under investigation. In breast cancer survivors with GSM, the application of 4% aqueous lidocaine prior to intercourse was found to result in significant reductions in pain associated with sexual activity and improvement in multiple sexual domains (*n* = 46) [87]. Male partners were also surveyed and reported no penile numbness. Hyaluronic acid is available without a prescription and one trial observed a significant decrease in vaginal pH of >6.5 from 30% of women to 19% of endometrial cancer survivors alongside significant improvements in vaginal health and sexual function as measured by the vaginal assessment scale (VAS), vulvar assessment scale (VuAS), and the female sexual function index (FSFI) [95]. The intervention was found to be useful in a separate study examining its use in menopausal women with the additional finding that frequency of use of 3–5 times weekly confers a greater benefit than twice weekly [93]. This relatively affordable and easy to find option is a reasonable treatment for women with contraindications to vaginal estrogen. Another study examined the use of hyaluronic acid combined with platelet concentrate in breast cancer survivors, however the study was limited by a small sample size (*n* = 20) and there was no control group receiving hyaluronic acid alone [88]. While participants noted an improvement in symptoms of vaginal dryness and dyspareunia, there was a 17% decrease in female sexual dysfunction (FSD) scores. It is unknown what, if any, role the addition of platelet concentrate had in the outcome.

While clinicians often recommend use of an over-the-counter lubricants and moisturizers during sexual activity, few studies have examined this class of unregulated products. Hickey et al. completed a randomized double-blind study of women with breast cancer with sexual pain and associated distress (*n* = 38) and found that participants experienced greater relief and satisfaction with the use of silicone lubricant versus water-based [89]. Twice as many women preferred silicone to water-based lubricants, however, 88% of women continued to experience clinically significant sexual distress regardless of lubricant type. Carter et al. evaluated female cancer survivors’ response and adherence to nonhormonal vaginal and sexual health treatments including education and instruction on the use of vaginal moisturizers and lubricants, pelvic floor exercises, dilator use, and psychosexual education regarding sexual changes associated with cancer treatment [23]. Their results (*n* = 169) showed excellent adherence with treatment indicating acceptability with 89% of women using a moisturizer 2–5 times weekly. Vaginal pH declined over time, indicating improved vaginal health. Sexual function scores increased, and participants expressed confidence about future sexual activity and a decrease in sexual concerns. Lee et al. provide evidence that further supports use of vaginal pH-balanced gel in their study that examined use of a vaginal pH-balanced gel for the control of atrophic vaginitis among breast cancer survivors and there was improvement in symptoms of dryness and dyspareunia in women who used the gel versus the placebo [92].

Lastly, because it is not uncommon for women to experience GSM and pelvic dysfunction concurrently, addressing both concerns together may improve efficacy and feasibility. Juraskova et al. examined a multimodal intervention (OVERcome) for breast cancer survivors, which consisted of the use of a vaginal moisturizer three times weekly, the use of olive oil as a lubricant during sexual activity, and pelvic floor muscle relaxation training [90]. Significant improvements in sexual function, dyspareunia, and quality of life were observed.

#### 3.3.2. Hormonal Topical Agents for Genitourinary Syndrome

Hormonal topical agents have been broadly evaluated in women at high risk for or with a personal history of breast cancer. A statement from The North American Menopause Society and the International Society for the Study of Women’s Sexual Health support consideration of the use of vaginal estrogen to treat symptoms of GSM that do not improve with nonhormonal therapy while highlighting the importance of shared decision making and collaboration with the patient’s oncology team [16]. A small study comparing vaginal estrogen and nonhormonal vaginal moisturizers in breast cancer survivors with GSM (*n* = 18) demonstrated superiority of twice weekly vaginal estrogen with sustained improvement in sexual function and vaginal health, although no significant effect on endometrial thickness and systemic estrogen levels was observed [84]. In contrast, there was no significant benefit in subjective or objective measures among women who used a vaginal moisturizer. Vaginal estrogen (estriol) in combination with lactobacilli was examined in one small study (*n* = 16) of postmenopausal breast cancer survivors on endocrine therapy [85]. Improvement was noted in symptoms of vaginal dryness and higher rates of sexual activity were observed, however, given there was a single arm in this small study, it is unknown to what, if any, impact the addition of lactobacilli had on outcomes.

Vaginal dehydroepiandrosterone (DHEA) has been FDA approved to treat the genitourinary syndrome of menopause in healthy women. At the cellular level DHEA is broken down into estradiol and testosterone, which both bind to estrogen and androgen receptors in the urogenital tract. A large (*n* = 464) RCT of menopausal women with a history of breast or gynecologic cancer was conducted and participants received either 3.25 mg DHEA, 6.5 mg DHEA, or a vaginal moisturizer [86]. All women reported improvement in vaginal symptoms at 12 weeks, however, only the 6.5 mg DHEA resulted in a significant improvement in overall sexual health as measured by the FSFI. Endometrial thickness and serum hormone levels were not assessed.

There is growing interest in the use of the oral SERM ospemifene, an estrogen agonist/antagonist that appears to have a negligible impact on breast and endometrial tissue but acts as an estrogen agonist in the urogenital tract. Rosa et al. examined the use of this medication in a group (*n* = 52) of young cervical cancer survivors with clinical symptoms of vulvovaginal atrophy [94]. After 6 months of treatment, there was significant improvement in scores on the vaginal health index, sexual function, body image, and sexual enjoyment. Further studies on breast and endometrial safety are necessary to use this therapy in women with a history of hormone receptor positive breast or gynecologic cancer.

#### 3.3.3. Fractional CO_2_ Laser Therapy for Genitourinary Syndrome

While not FDA approved to treat genitourinary syndrome of menopause at this time, the use of a fractional CO_2_ laser has been examined as a nonhormonal therapy for GSM, with several studies showing promise. Gittens and Mullen performed a retrospective chart review of women with a history of breast cancer on endocrine therapy who had treatment with fractional microablative CO_2_ laser therapy [78]. Participants experienced significant improvement across domains of sexual function, sexual distress, and the Wong–Baker faces scale. Further, there was improvement in both menopausal women and women with a history of breast cancer treated with endocrine therapy. While promising, this study was limited by small sample size (*n* = 25) and by a retrospective design. Angioli et al. examined the use of CO_2_ laser therapy in women with previous gynecologic or breast cancer (*n* = 165) [79]. In this retrospective study, statistically significant improvements were noted in the domains of dryness, dyspareunia, burning, pain at the introitus, and itching [79]. Long-term data from Pieralli et al. demonstrated improvement in dyspareunia 11 months after treatment in breast cancer survivors (*n* = 50), suggesting that this therapy may offer long term relief of bothersome GSM symptoms [80]. Beyond improved vulvovaginal symptoms in breast cancer survivors, Becorpi et al. demonstrated alterations in inflammatory and modulatory cytokines that may support the underlying pathophysiology of epithelial remodeling after CO_2_ laser therapy [75]. Several other studies reviewed in breast cancer patients reported similar improvement in symptoms and acceptability of this treatment, however large RCTs are lacking, particularly in women with cancer [77,81,82] Concerns with CO_2_ laser therapy include post-procedure pain and bleeding and consensus on the optimal number of cycles to administer and need/frequency for retreatment [83].

#### 3.3.4. Educational Programs for Genitourinary Syndrome in Women with Cancer

Finally, the utility of a web-based women’s health survivorship care plan for young breast cancer survivors with sexual health concerns was assessed in a RCT of 184 patients [76]. Over 24 weeks, the intervention arm having access to a personalized web-based survivorship care plane, relevant education and online clinical guidance for patients, and text messages with sexual health tips. The control arm received access to the curated web-based resources and study adherence text messages only. The results of this novel trial showed that vaginal symptoms associated with breast cancer therapies were improved in the arm enrolled on the online survivorship care plan [76].

#### 3.3.5. Summary: Interventions to Address GSM

In summary, while there are several options available to treat bothersome symptoms of GSM that often result in sexual pain, further study is needed in many areas. The CO_2_ laser is appealing to many patients and clinicians given the nonhormonal nature and potential for sustained improvement in vaginal tissue over time. However, large-scale randomized placebo-controlled trials are necessary to earn FDA approval and validate its use and safety in this population and in the general population. The oral SERM ospemifene represents a novel approach to treatment and while preclinical trials suggest antiestrogenic effects in the breast, studies of cancer survivors are needed to establish a clear safety record in this patient population [16]. Until more robust data is available, clinicians must continue to engage women in shared decision-making, education, and collaboration with the patient’s oncology care team.

### 3.4. Pelvic Floor Dysfunction in Women after Cancer

Cancer survivors who undergo pelvic surgery or pelvic radiation are at a unique risk for pelvic floor dysfunction as a result of injury to organs and vessels, psychologic response, and social/relational experiences [117]. Injury to tissue can include fibrosis, inflammation, endothelial damage, inflammation, ischemia, changes in vaginal discharge, and more. Beyond physical changes, and regardless of treatments/exposures, changes in psychosexual and social/relational domains may result in pelvic floor dysfunction in cancer survivors. These changes may be detrimental to sexual health.

#### Pelvic Floor Rehabilitation and Dilator Use for Female Cancer Survivors

Yang et al. evaluated a pelvic rehabilitation program in gynecologic cancer survivors, consisting of an exercise training session with biofeedback and core strengthening, in combination with weekly counseling, which resulted in significant improvement in pelvic floor strength and numerous sexual function domains (*n* = 24) [97]. Similar findings, again for gynecologic cancer survivors, were observed by Cyr et al. [98]. Additionally, this study assessed feasibility and satisfaction and found excellent adherence (88%) and patient satisfaction, with 90% of participants reporting “much” or “very much” improvement. An evaluation of independent use of vaginal dilators among colorectal cancer survivors who had undergone pelvic radiation was examined by Law et al. [96]. This study reported poor adherence to therapy over time with just 25% adherence in the fourth quarter of the study, suggesting that women may do their best when part of an in-person pelvic therapy program.

As previously mentioned, Juraskova et al. tested a multimodal intervention for breast cancer survivors, combining vaginal moisturizer and lubricant use with pelvic floor muscle relaxation training to prevent pelvic floor hypertonus (at 0 and 4 weeks, with home exercise twice daily and follow-up at 12 and 26 weeks) [90]. This results in improved function, dyspareunia, and quality of life being observed.

### 3.5. Vasomotor Symptoms of Menopause in Women with and Surviving Cancer

Vasomotor symptoms associated with menopause, such as hot flashes and night sweats, are common symptoms associated with certain cytotoxic or antihormonal therapies and may have a detrimental effect on patients’ sexual health, sleep quality and quantity, and sexual self and body image [109]. A number of interventions have been trialed to improve vasomotor symptoms, these are detailed below and summarized in Table 2.

#### 3.5.1. Pharmaceutical Agents for Vasomotor Symptoms of Menopause

In clinical practice, selective serotonin reuptake inhibitors (SSRIs) or serotonin-norepinephrine reuptake inhibitors (SNRIs) are used to reduce the frequency or severity of hot flashes as they have shown efficacy in women without cancers experiencing vasomotor symptoms associated with menopause. Several studies report this practice. For example, in a randomized, double-blind, placebo-controlled crossover study of sertraline (SSRI) for the treatment of hot flashes in women with early-stage breast cancer on tamoxifen, hot flash frequency decreased in 50% in the patients on sertraline as compared with the placebo [105]. Likewise, paroxetine (SSRI) was studies in a RCT for patients with gynecologic cancers and showed a similar benefit with a significant reduction in hot flashes and nighttime awakenings from night sweats [116]. Importantly, while SSRIs and SNRIs may be beneficial for hot flashes, they also may result in diminished sexual desire and increased latency to orgasm; therefore, in addressing hot flashes as they relate to sexual function, weighing risks and benefits is critical. Further, additional assessing effects on sexual function in this setting would be beneficial. Unlike SSRIs and SNRIs, bupropion, an atypical antidepressant, does not affect sexual function. In the breast cancer patient population, bupropion was studied in a randomized phase II double-blind crossover study enrolling 55 women who at baseline experienced seven or more hot flashes per week [106]. Interestingly, bupropion reduced the hot flashes by 1.26% per day and the ‘hot flash score’ by 6.31% whereas the placebo reduced hot flashes by 2.11% per day and the ‘hot flash score’ by 30.75%, thus showing no benefit of the addition of bupropion for this study.

In addition to antidepressant medications, the use of a stellate-ganglion block was used in a prospective trial of 13 breast cancer survivors with severe hot flashes and night awakenings [107]. Patients recorded their hot flashes in a daily diary and by use of the hot flash score and night awakenings by use of the Pittsburgh sleep quality index weekly for 12 weeks. The findings showed that there were no adverse events in this small patient sample with the use of a stellate-ganglion block and, even despite the small sample size, there was a significant decrease in the total number of hot flashes and night awakenings during the duration of the study.

#### 3.5.2. Psychoeducational and Psychotherapeutic Programs to Address Vasomotor Symptoms of Menopause after Cancer

Perhaps the most promise in the reduction of vasomotor symptoms for cancer survivors is in the utilization of cognitive behavioral therapy (CBT). There have been several studies, including a small pilot study of a guided, internet-based CBT program with self-report questionnaires in 21 breast cancer survivors with menopausal symptoms [112]. A total of 90% of the participants completed the program, and importantly there was a significant decrease in overall hot flashes and night sweats reported in the study. The results of this study prompted additional trials by the same investigator team using internet-based CBT for treatment-induced menopausal symptoms in breast cancer survivors [110]. In this trial, 254 patients were randomized to therapist guided versus self-managed internet-based cognitive behavioral therapy or to a waiting list control group. Compared to the control group, the guided and self-managed groups reported a significant decrease in the impact and frequency of hot flashes and night sweats (*p* < 0.001) [110,111]. The same group of investigators published a subsequent study evaluating the cost-utility of an internet-based cognitive behavioral therapy program for breast cancer survivors, showing that this approach is more cost-effective compared to the traditional model with a lower impact on healthcare costs [113]. A similarly designed RCT of a six-week, in-person, group CBT program for women with vasomotor symptoms after breast cancer treatment showed similarly promising results with a significant reduction in hot flashes and night sweats in the CBT arm [108]. A separate online psychoeducation program was tested in a randomized study of 91 Asian American breast cancer survivors, with decreased distress of total menopausal symptoms over time; subscales (physical, psychological, and psychosomatic) trended toward an effect but failed to reach significance [115].

Beyond traditional CBT-based interventions, additional mindfulness-based practices, such as paced breathing and hypnosis, have been evaluated. For example, paced respiration is sometimes recommended for vasomotor symptom management despite limited empirical evidence. In a 16-week, 3-group, partially blinded, controlled trial with a 2:2:1 randomization, 218 patients (96 with a history of breast cancer) were assigned to the intervention (paced breathing at time of the hot flash, instruction with disc or booklet) versus fast shallow breathing control, versus usual care control [114]. There were no significant differences between the three groups. A separate small (*n* = 16) prospective pilot study evaluated the use of hypnosis for the treatment of hot flashes for breast cancer patients, the results indicated a 59% decrease in the total daily hot flashes and a 70% decrease in weekly hot flash scores from their baselines [109]. In contrast to the benefit demonstrated from acupuncture and hypnosis, albeit in small studies, homeopathy and paced breathing have been less promising.

#### 3.5.3. Educational Programs to Address Vasomotor Symptoms of Menopause after Cancer

A previously described study (Section 3.3.4) assessed the utility of a web-based women’s health survivorship care plan for young breast cancer survivors. The results of this novel trial showed that menopausal symptoms associated with breast cancer therapies were improved in the arm enrolled on the online survivorship care plan [76].

#### 3.5.4. Exercise Programs to Address Vasomotor Symptoms of Menopause after Cancer

Two small RCTs (*n* = 40 and *n* = 37) assessing yoga and meditation as modalities to reduce menopausal symptoms in breast cancer survivors have shown significant improvements in lowering these symptoms as compared with placebo control groups over time frames spanning 8 weeks up to 3 months [103,104]. In the same study, Carson et al. also showed a reduction in the level of joint pain, fatigue, sleep disturbances, and symptom-related bother and vigor [104].

#### 3.5.5. Complementary and Alternative Medicine Practices to Address Vasomotor Symptoms of Menopause after Cancer

Integrative practices, such as acupuncture and herbal or nutritional supplements, have been evaluated for their use for vasomotor symptoms of menopause. The use of acupuncture to reduce sleep disturbance and hot flashes in postmenopausal breast cancer patients was studied in a prospective trial showing encouraging results with a decrease in hot flashes in the acupuncture group (*p* = 0.02, *n* = 10 patients) [99]. One small RCT assessing the use of homeopathic supplements (amyl nitrate, *Sanguinaria canadensis*, and *Lachesis*) for menopausal symptoms in breast cancer survivors failed to show a benefit in comparison to the placebo in the primary outcome measure, which was the hot flash severity score, however a statistically significant improvement was found after one year [100].

While there is anecdotal evidence to support herbal supplements in reducing hot flashes in perimenopausal women, caution needs to be taken for similar agents in our cancer survivor populations as they may worsen pre-existing symptoms or provide estrogenic effects, which could counteract antiendocrine therapies. The use of St. John’s wort (*Hypericum perforatum*) was studied in a RCT of 47 women (mainly breast cancer survivors) with 12 weeks of treatment showing no significant difference in improvement but with a significantly improved outcome after three months [101]. Similarly, an RCT trial of oral soy supplements versus the placebo for the treatment of vasomotor symptoms in breast cancer patients has been conducted, however, in this study there was no significant benefit to this supplement [102]. As there may be an interaction with cancer therapies, it is always important to encourage patients to discuss the supplements that they are taking with their medical providers.

#### 3.5.6. Summary: Interventions to Address Vasomotor Symptoms of Menopause

Several studies have aimed to evaluate and treat vasomotor symptoms of menopause in cancer survivors. SSRIs, cognitive behavioral therapy, and acupuncture have been the most successful in addressing this issue, which often is associated with a significant reduction in patients’ quality of life and sexual health. Importantly, all studies identified in this review were focused on survivors of breast cancer; studies addressing treatment of these symptoms in women surviving other cancers are needed. Further, it is important to continue to evaluate multiple modalities ranging from pharmaceutical options to complementary therapies as patients’ symptoms and expectations vary.

## 4. Discussion

Despite affecting roughly 60% of female cancer survivors across their life span, sexual health concerns are vastly under-recognized and undertreated [1,2,3,4,10,11,12]. Barriers to care include limited access to widely available sexual health interventions and a lack of provider knowledge of existing treatments for sexual function concerns [11,13]. This scoping review aims to serve as a resource to providers and researchers alike, summarizing the current literature for interventions for sexual function concerns in female cancer survivors while identifying gaps in evidence.

Conclusions about interventions for each type of sexual function concern are drawn by the section above. The mostly widely studied intervention type to address both sexual function and body image concerns was multicomponent psychoeducational and psychotherapeutic programming, aimed at the individual cancer survivor (vs. partner-based interventions). Comparison across interventions is limited by a wide variation in study design, sample size, duration of follow-up, and outcome criteria. Importantly, many of these studies had results that were encouraging for this intervention type, with various approaches showing benefit. However, because these interventions often consisted of multiple components, it is impossible to understand which components were the most impactful and should be included as sexual medicine programs are developed in practice. This is particularly true for psychoeducational interventions in which, beyond intervention components, interventions were conducted by a wide range of individuals such as psychologists, sex therapists, nurses, mid-wives, social workers, and peer counselors. These individuals vary greatly in training and availability to patients seen at cancer centers and community settings in the US and abroad. However, several studies did suggest that the training background of providers may not be as significant, with nurse-led and peer-led interventions at times resulting in similar outcomes to specialist-led interventions [41,42,54]. Lastly, while there was a significant range in when during and after therapy interventions were offered, it is unknown if there is an “ideal” time to address sexual health concerns among women with cancer; studies comparing timing of interventions would be informative. Additional information on effective components, intensity or scope of the program, and facilitators is vital for the design, implementation, and accessibility of interventions to prevent, minimize risk for, or treat sexual health concerns. Further, because sexual health is affected not only by biomedical and psychological aspects, but also by social/relational aspects, further research evaluating the utility of patient–partner focused interventions is also warranted.

The studies we reviewed focused on improving sexual health in women with breast, gynecologic, colon, and anal cancers. This is expected because the first three are the most common forms of cancer in women in the US and several other countries. In addition, these cancers and their treatments are well known to adversely impact sexual health. It is essential, however, that we also better understand the sexual health concerns of young adults and women with other types of cancer as well such as lymphoma, sarcoma, blood, lung, and skin cancers. Furthermore, studies of sexual health in women with breast cancer typically restricted their inclusion criteria to individuals with Stage I-III disease. There is a paucity of both qualitative and quantitative data on sexual health concerns among women with metastatic or advanced cancers.

Finally, very few of the investigations analyzed the impact of the sexual health interventions for women with cancer in different age groups. Most studies included middle-aged women coping with cancer. While Ganz et al. and others note higher rates of distress related to sexual health concerns in younger women with breast cancer, only a few studies were able address the needs of younger individuals [28,35,56,60,61,63,76,94]. In addition, investigations in the United States predominately included White women, except for Schover et al.’s study with Black women with breast cancer [42]. The lack of ethnic diversity in study samples continues to be unacceptable and comprises generalizability of findings to varied populations of women living with cancer survivorship challenges. Many investigations also focused on partnered women; we have little data on the sexual health concerns of single or unpartnered women with cancer irrespective of their dating interests. Few studies mentioned the participant’s gender identity, the sex or gender of the partners, or if they included participants with cancer from the trans community or individuals with non-binary gender identities.

Beyond the limitations of the existing literature, this scoping review has several limitations. First, this review did not seek to formally evaluate the quality of evidence, including a wide range of study designs, methods, interventions, and outcomes. As such, this review serves to summarize existing literature but cannot yield specific results or draw conclusions regarding specific recommendations for interventions for sexual health concerns.

## 5. Conclusions

Further research is critical to fill in the many aforementioned gaps in knowledge. Despite this, the studies assessed in this review lay important groundwork that should be used by providers to develop patient resources, and cancer centers to implement multidisciplinary sexual health care, for female cancer survivors. As it is well-documented that sexual health concerns are under-recognized and under-met among cancer survivors, it is critical for both clinicians and researchers to become more aware of these concerns and options for addressing them [3,118,119,120]. Increasing access to sexual health care through such interventions and addressing insurance reimbursement for these services, is critical to improving health equity and quality of life for all cancer survivors.

## Figures and Tables

**Table 1:** Interventions for general sexual function and body image concerns.

Patient Populations	Intervention Type	Reference Number(s)
**General sexual function concerns**		
All adult cancers	Multimodal clinic	[22,23,24]
	Complementary/alternative medicine	[25]
	Psychoeducation (technology-based)	[26]
	Testosterone replacement	[27]
AYA patients	Psychoeducation (In-person, individual)	[28]
Breast cancer	Exercise	[29,30,31,32]
	Psychoeducation/biomedical program (In-person, group)	[33]
	Psychoeducation (In-person, group)	[34,35,36,37,38]
	Psychoeducation (In-person, individual)	[39,40]
	Psychoeducation (technology-based)	[41,42,43,44,45,46,47,48]
Cervical cancer	Psychoeducation/biomedical program (In-person, group)	[49]
Colorectal cancer	Psychoeducation (technology-based)	[50]
Endometrial cancer	Exercise	[51]
Gynecologic cancers (all)	Multimodal clinic	[52]
	Psychoeducation (In-person, individual)	[40,50,53,54]
	Psychoeducation (technology-based)	[48]
HSCT recipients	Multimodal clinic	[55]
Ovarian cancer	Psychoeducation/biomedical program (In-person, group)	[56,57]
	Psychoeducation (In-person, group)	[58]
Rectal and anal cancer	Psychoeducation (In-person, individual)	[59]
**Body image concerns**		
AYA patients	Psychoeducation (In-person, individual)	[28]
	Psychoeducation (technology-based)	[60]
	Social/expressive programming	[61,62,63]
Breast cancer	Cosmetic programming	[64]
	Exercise	[29,32,65]
	Psychoeducation/biomedical program (In-person, group)	[33]
	Psychoeducation (In-person, group)	[37,66,67]
	Psychoeducation (In-person, individual)	[40]
	Psychoeducation (technology-based)	[47,68]
Gynecologic cancers (all)	Psychoeducation (In-person, individual)	[40]
	Psychoeducation (technology-based)	[68]
Head/neck cancers	Cosmetic programming	[69]
	Psychoeducation (technology-based)	[70]

**Table 2:** Interventions to address genitourinary syndrome, pelvic floor dysfunction, and vasomotor symptoms of menopause in female cancer survivors.

Patient Populations	Intervention Type	Reference Number(s)
**Genitourinary syndrome**		
All adult cancers	Non-hormonal topical agent	[23]
Breast cancer	Education	[76]
	Er:YAG laser therapy	[77]
	Fractional CO_2_ laser therapy	[75,78,79,80,81,82,83]
	Hormonal topical agents	[84,85,86]
	Non-hormonal topical agent	[87,88,89,90,91,92,93]
Cervical cancer	Hormonal topical agents	[94]
Endometrial cancer	Non-hormonal topical agent	[93,95]
Gynecologic cancers (all)	Fractional CO_2_ laser therapy	[79]
	Hormonal topical agents	[86]
**Pelvic floor dysfunction**		
Breast cancer	Pelvic floor rehabilitation	[90]
Colorectal cancer	Dilator therapy	[96]
Gynecologic cancers (all)	Dilator therapy	[96]
	Pelvic floor rehabilitation	[97,98]
**Vasomotor symptoms of menopause**		
Breast cancer	Complementary/alternative medicine	[99,100,101,102]
	Education	[76]
	Exercise	[103,104]
	Pharmacologic agents	[105,106,107]
	Psychoeducation (In-person, group)	[108]
	Psychoeducation (In-person, individual)	[109]
	Psychoeducation (technology-based)	[110,111,112,113,114,115]
Gynecologic cancers (all)	Pharmacologic agents	[116]

## Data Availability

The search strategy is available in the Supplementary Materials.

## Supplementary Materials

The search strategy used for this review is available online at https://www.mdpi.com/article/10.3390/cancers13133153/s1, Table S1 PubMed search strategy.
