# Supplementary material for: Interventions to Improve Sexual Health in Women Living with and Surviving Cancer: Review and Recommendations
Source: Cancers (Basel). 2021 Jun 24;13(13):3153. doi: 10.3390/cancers13133153 (PMC8268945; doi:10.3390/cancers13133153)
Supplement: Supplementary file 1 [file cancers-13-03153-s001.zip › cancers-1206439-supplementary.pdf]

cancers

Supplementary Material: Interventions to Improve Sexual Health in Women Living with and Surviving Cancer: Review and Recommendations

Jenna Sopfe, Jessica Pettigrew, Anosheh Afghahi, Leslie C. Appiah and Helen L. Coons

Table S1. PubMed search strategy.

Concepts	Cancer/Survivors	Survivors/Survivorship	Sexuality/Sexual Function/Sexual Health
Entry Terms and Keywords (Title/Abstract)	Neoplasm[tiab] OR Neoplasms[tiab] OR Cancer[tiab] OR Cancers[tiab] OR Malignancy[tiab] OR Malignancies[tiab]OR	Survivors[tiab] OR Survivor[tiab] OR Survivorship[tiab] OR "cancersurvivor"[tiab] OR "cancer survivors"[tiab] OR "cancer survivorship" OR "lateeffects"[tiab] OR "long-term outcomes"[tiab]OR	"sexual"[tiab] OR "Sex Orientation"[tiab] OR Sexuality[tiab] OR Orgasms[tiab] OR "SexDevelopment"[tiab] OR "SexualDevelopment"[tiab] OR "Psychological SexualDysfunction"[tiab] OR "Psychological Sexual Dysfunctions"[tiab] OR Psychosexual[tiab]OR Psycho-sexual[tiab] OR "OrgasmicDisorder"[tiab] OR "Orgasmic Disorders"[tiab] OR "Sexual Arousal Disorder"[tiab] OR "Sexual Arousal Disorders"[tiab] OR "Dyspareunia"[tiab] OR "SexDisorders"[tiab] OR Libido[tiab] OR "Vaginalatrophy"[tiab] OR "Gonadotoxicity"[tiab] OR "Gonadal dysfunction"[tiab] OR "vaginalstenosis"[tiab] OR "Bodyimage"[tiab] OR "BodyImages"[tiab] OR "BodySchema"[tiab] OR "BodySchemas"[tiab] OR "SexualPartner"[tiab] OR "SexualPartners"[tiab] OR "romanticrelationships"[tiab] OR "sexualintimacy"[tiab] OR
MeSH (Medical Subject Headings)	"Neoplasms"[Mesh]	"Cancer Survivors"[Mesh]	"Sexual Behavior"[Mesh]OR"Orgasm"[Mesh]OR "Sexual Development"[Mesh]OR"Psychosexual Development"[Mesh]OR "Sexual Dysfunctions, Psychological"[Mesh]OR"Sexual Dysfunction, Physiological"[Mesh]OR"Libido"[Mesh] OR"Body Image"[Mesh] OR"Sexual Partners"[Mesh]
